# Adaptation of soil microbial growth to temperature: Using a tropical elevation gradient to predict future changes

**DOI:** 10.1111/gcb.14502

**Published:** 2019-01-06

**Authors:** Andrew T. Nottingham, Erland Bååth, Stephanie Reischke, Norma Salinas, Patrick Meir

**Affiliations:** ^1^ School of Geosciences University of Edinburgh, Crew Building, Kings Buildings Edinburgh EH9 3FF United Kingdom; ^2^ Smithsonian Tropical Research Institute 0843-03092 Balboa Ancon Panama; ^3^ Department of Biology, Section of Microbial Ecology Lund University Lund Sweden; ^4^ Instituto de Ciencias de la Naturaleza, Territorio y Energías Renovables Pontificia Universidad Catolica del Peru, Av. Universitaria 1801 San Miguel Lima 32 Peru; ^5^ Research School of Biology Australian National University Canberra Australian Capital Territory 2601 Australia

**Keywords:** bacteria, climate warming, fungi, *Q*_10_, Ratkowsky equation, soil carbon cycle, tropical forest

## Abstract

Terrestrial biogeochemical feedbacks to the climate are strongly modulated by the temperature response of soil microorganisms. Tropical forests, in particular, exert a major influence on global climate because they are the most productive terrestrial ecosystem. We used an elevation gradient across tropical forest in the Andes (a gradient of 20°C mean annual temperature, MAT), to test whether soil bacterial and fungal community growth responses are adapted to long‐term temperature differences. We evaluated the temperature dependency of soil bacterial and fungal growth using the leucine‐ and acetate‐incorporation methods, respectively, and determined indices for the temperature response of growth: *Q*_10_ (temperature sensitivity over a given 10oC range) and *T*_min _(the minimum temperature for growth). For both bacterial and fungal communities, increased MAT (decreased elevation) resulted in increases in *Q*_10 _and *T*_min_ of growth. Across a MAT range from 6°C to 26°C, the *Q*_10 _and *T*_min_ varied for bacterial growth (*Q*_10–20_ = 2.4 to 3.5; *T*_min_ = −8°C to −1.5°C) and fungal growth (*Q*_10–20_ = 2.6 to 3.6; *T*_min_ = −6°C to −1°C). Thus, bacteria and fungi did not differ significantly in their growth temperature responses with changes in MAT. Our findings indicate that across natural temperature gradients, each increase in MAT by 1°C results in increases in *T*_min_ of microbial growth by approximately 0.3°C and *Q*_10–20 _by 0.05, consistent with long‐term temperature adaptation of soil microbial communities. A 2°C warming would increase microbial activity across a MAT gradient of 6°C to 26°C by 28% to 15%, respectively, and temperature adaptation of microbial communities would further increase activity by 1.2% to 0.3%. The impact of warming on microbial activity, and the related impact on soil carbon cycling, is thus greater in regions with lower MAT. These results can be used to predict future changes in the temperature response of microbial activity over different levels of warming and over large temperature ranges, extending to tropical regions.

## 1 INTRODUCTION

Soil microorganisms regulate terrestrial biogeochemical cycles, and their response to temperature is a critical factor in regulating feedbacks associated with climate warming (Davidson & Janssens, 2006). Models have demonstrated that the nature of temperature‐adaptive responses in soil microbial physiology, community composition, enzyme function or growth, may have major influences on atmospheric CO_2_ accumulation in the 21st century (Wieder, Bonan, & Allison, 2013). Of all terrestrial ecosystems, tropical forests exert the largest influence on global climate because they are the most productive and have the highest respiration rates (Beer et al., 2010; Pan et al., 2011), in addition to containing the highest biomass of soil microorganisms (Serna‐Chavez, Fierer, & Bodegom, 2013). It is surprising, therefore, that we have a limited understanding of the temperature response of soil microbial communities in these ecosystems.

Research on the temperature response of soil organic matter cycling has been extensive, albeit concentrated outside the tropics, but a consensus remains elusive (Conant et al., 2011; Karhu et al., 2014; Kirschbaum, 2006). The focus of this work has often been on the temperature response of respiration, in the context of its potential impact as a positive feedback on climate warming (Davidson & Janssens, 2006) and the potential for a temperature‐adaptive response of the microbial community in affecting this feedback (Bradford et al., 2008; Karhu et al., 2014). Such an adaptation response has been defined as a change in microbial community composition, physiology or enzyme function, which has a net result of metabolism being better‐optimized to a given temperature (Bárcenas‐Moreno, Gomez‐Brandon, Rousk, & Bååth, 2009; Bradford, 2013). The lack of consensus among studies on the temperature response of microbial activity arises partly because respiration, a commonly measured index of microbial activity, has an “apparent” temperature sensitivity that is influenced by multiple environmental variables (“indirect effects”) that vary among soils (Nottingham, Whitaker, et al., 2015). It will also be affected indirectly by factors other than the temperature regime, such as substrate availability and moisture (Davidson & Janssens, 2006; Nottingham, Whitaker, et al., 2015). To isolate the direct effect of temperature, one must estimate the “intrinsic” temperature sensitivities of specific processes such as carbon‐assimilation, enzyme activities and growth, which are independent of these indirect effects. These intrinsic temperature sensitivities can be assessed in controlled short‐term incubation experiments (Kirschbaum, 2006), providing standard reproducible information on microbial temperature responses which are then comparable across biomes.

Another reason for the lack of consensus is due to the different ways in which temperature responses are modelled. The temperature sensitivity of microbial processes, such as growth and respiration, has been described using various metrics. A commonly used parameter is the *Q*_10_ value, which represents the ratio of a process at (*T* + 10°C)/*T*, where *T* = standard reference temperature. However, comparison of *Q*_10_ among studies requires careful consideration of the differences in temperature range and reference temperatures used for its calculation, because *Q*_10_ is not constant over a given temperature range. The *Q*_10_ of respiration and microbial growth is higher when determined at lower temperatures (Bååth, 2018; Kirschbaum, 2006; Lloyd & Taylor, 1994) and models often incorporate a higher *Q*_10_ for lower temperatures (Del Grosso et al., 2005; Jenkinson, Adams, & Wild, 1991). This dependency of *Q*_10_ on the measurement temperature range makes it difficult to compare* Q*_10_ among studies that use different temperature ranges in their calculations. The measurement of *Q*_10_ over a large temperature range, assuming constant *Q*_10_, can also introduce problems in predicting the effects of temperature on respiration and growth (Bååth, 2018). Temperature dependency is also often modelled using an Arrhenius relationship, $k=Ae^{\left[E_{\text{a}}/\text{RT}\right]}$, where *k* = rate, *A* = constant, *E*_a _= activation energy, R = universal gas constant, and *T* = absolute temperature. Here, the activation energy (*E*_a_) determines the temperature sensitivity. However, since there is a close relationship between *E*_a_ and *Q*_10_ within the range of temperatures normally found in soils (Raven & Geider, 1988), *E*_a_ has the same problems in interpretation and determination as *Q*_10_.

**Figure 1:**
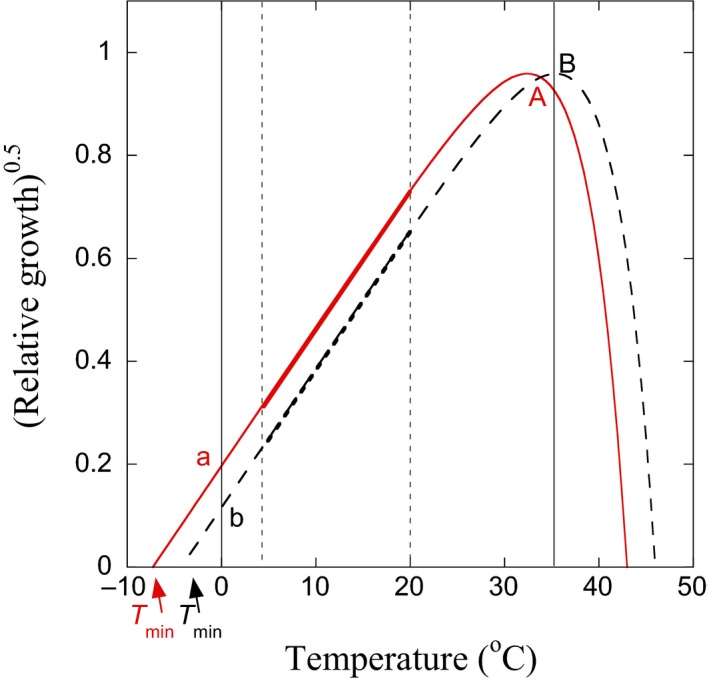
A comparison of the temperature sensitivity of growth for two hypothetical microbial communities, plotted with a square root transformation. Community A (full line, red) is low temperature‐adapted (*T*_min _= −7.3°C), and community B (dashed line, black) is high temperature‐adapted (*T*_min _= −4.3°C). Three indices of temperature sensitivity are shown. *T*_min_ was determined by linear regression (thick lines) using measurements at 20 and 4°C (thin stippled vertical lines) and extrapolation, where *T*_min_ for community A < *T*_min_ for community B. *Q*_10–20 _(see Methods) was calculated using the same regression, where *Q*_10–20_ for community A <*Q*_10–20_ for community B. A temperature sensitivity index (log 35/0) was determined by the log of the ratio of growth at 35°C and 0°C (thin vertical lines), that is log [(A/a)^2^] for community A and log [(B/b)^2^] for community B. The temperature sensitivity index log 35/0 for community A <log 35/0 for community B [Colour figure can be viewed at wileyonlinelibrary.com]

An alternative approach that can be used to characterize respiration and growth, while below its temperature optimum (*T*_opt_), is the use of the square root relationship: *A* ^0.5 ^= *a × *(*T*–*T*_min_), where *A* is activity (e.g., growth or respiration), *T*_min_ the apparent minimum temperature for activity (°C), and *a *is a slope parameter related to absolute activity (Ratkowsky, Olley, Mcmeekin, & Ball, 1982) (Figure 1). The square root relationship, also called the Ratkowsky equation, has been widely used to model the rate of bacterial growth in water (Bell & Ahlgren, 1987; Li & Dickie, 1987) and bacterial and fungal growth in soil (Dıaz‐Raviña, Frostegård, & Bååth, 1994; Pietikäinen, Pettersson, & Bååth, 2005; Rinnan, Rousk, Yergeau, Kowalchuk, & Bååth, 2009; van Gestel, Reischke, & Bååth, 2013). It has also been shown to be an adequate representation of the temperature responses of respiration and decomposition (Bååth, 2018; Kätterer, Reichstein, Andren, & Lomander, 1998; Pietikäinen et al., 2005), and it has been shown that *T*_min_ increases and decreases following community temperature adaptation to the thermic environment (Bååth, 2018; Bárcenas‐Moreno et al., 2009). Because it is independent of the temperature range at which it is calculated, it can be compared more easily among studies than *E*_a_ or *Q*_10_. By determining *T*_min_ following the square root model, we can obtain information on the temperature responses of microbial growth or respiration, which can be related with other process rates such as enzyme kinetics (*E*_a_, *Q*_10_). The *T*_min_ metric, therefore, provides information on the community‐level adaptation to temperature, which can be easily compared across biomes and can be used to predict future effects of climate change.

Biogeographic variation in the *T*_min_ for microbial growth and respiration in soils from different ecosystems has been tentatively estimated as −10 to −15°C in Arctic/Antarctic regions, −5 and −10°C in temperate regions and 0 to −5°C in tropical regions (Pietikäinen et al., 2005; Rinnan et al., 2009; van Gestel et al., 2013). Furthermore, by combining several studies on the effect of mean annual temperature (MAT) on *T*_min_ for bacterial growth, it has been predicted that a 1°C increase in MAT would result in an increased *T*_min_ of between 0.2 and 0.3°C (Rousk, Frey, & Bååth, 2012). Bååth (2018) also predicted, using a tentative global envelope of *T*_min_ in soils, that a 1°C increase in MAT would result in an increased *T*_min_ of around 0.3°C. However, the temperature sensitivity of soil microbial growth has been studied across only a limited MAT range. For example, Rinnan et al. (2009) studied soils ranging from −4°C to +9°C, while Rousk et al. (2012) compared a MAT of 7°C with an artificially heated treatment with a MAT of 12°C. To be able to predict changes in temperature sensitivity over a major part of the global MAT variation (i.e., −15 to 30°C), a larger range needs to be tested, of course also including tropical regions.

The temperature sensitivity of bacterial growth in soil has been reasonably well studied (Rinnan et al., 2009; Rousk et al., 2012; van Gestel et al., 2013), but this is not the case for fungal growth. Only two earlier studies, in soils and only from the temperate zone, have compared *T*_min_ for fungi and bacteria (Birgander, Olsson, & Rousk, 2018; Pietikäinen et al., 2005). Similar but slightly lower *T*_min_ for fungal compared to bacterial growth was found, suggesting fungi to be better adapted to low temperature conditions. However, more studies are needed to test this further, covering a larger variation in MAT.

Elevation gradients on mountainsides have been used to understand plant biogeography by ecologists since the 18th century (Linnaeus, 1781; von Humboldt & Bonpland, 1805), but more recently they have been used as powerful tools to understand how climate change affects plant and microbial ecology (Nottingham, Whitaker, et al., 2015; Sundqvist, Sanders, & Wardle, 2013), by revealing the long‐term temperature acclimation or adaptive changes in plant physiology, soil processes and soil microbial composition (Giardina, Litton, Crow, & Asner, 2014; Girardin et al., 2014; Nottingham, Fierer, et al., 2018). Here, we used a 3.5 km elevation gradient in Peru to explore the long‐term temperature adaptation of bacterial and fungal growth to a 20°C gradient in MAT. We tested the following hypotheses: (a) Increasing MAT (i.e., decreasing elevation) will increase the temperature optima for bacterial growth, resulting in higher *T*_min_, temperature sensitivity index (growth at 35°C/0°C) in lower elevation sites with higher MAT; (b) *T*_min_ will increase around 0.2 to 0.4 per degree Celsius increase in MAT, equivalent to around 0.05 higher *Q*_10–20_ per degree increase in MAT; and (c) based on earlier results in temperate‐zone studies (Pietikäinen et al., 2005), we hypothesize a lower temperature sensitivity index for fungi than bacteria. Since our results cover a gradient in MAT from 6 to 26°C, our data can be used to predict future changes in the temperature sensitivity of microbial growth and activity in soil over a large range of MAT.

## 2 MATERIALS AND METHODS

### 2.1 Study sites

The elevation transect under study lies on the Eastern flank of the Andes in southeastern Peru, in the upper Madre de Dios/Madeira watershed. The transect is approximately 270 km in length and spans 3,450 m in elevation from 194 m to 3,644 m above sea level (asl). The transect consists of 14 sites, each with a 1 ha permanent sampling plot, all in old growth tropical forest except for one site on high elevation grassland (Supporting Information Table S1).

Mean annual temperature (MAT) decreases with increasing elevation across the transect (dropping from 26 to 6°C; Supporting Information Figure S1). There is little variation in seasonal temperature across the gradient, with mean daily air temperature differing only by about 4°C between warmest and coolest month, irrespective of elevation, although diurnal variation can increase this range slightly (Rapp & Silman, 2012). Mean annual precipitation (MAP) is consistently high and does not vary consistently with elevation, ranging from 1,506 to 5,302 mm/year among the sites (Nottingham, Whitaker, et al., 2015).

The plots are situated on predominantly Palaeozoic (~450 Ma) meta‐sedimentary mudstone (~80%), with plutonic intrusions (granite) underlying the sites between 1,500 and 2,020 m asl. The soils at the sites above 2,520 m are Umbrisols (Inceptisols), while the soils from 1,000 to 2,020 m are Cambisols (Inceptisols). The soils below 1,000 m, at the two lowland sites, are HaplicAlisols (Ultisols) (194 m asl) and Haplic Cambisols (Inceptisols) (210 m asl) (according to FAO, with USDA Soil Taxonomy in parentheses). Further descriptions of soil, climate and floristic composition of these sites are reported elsewhere (Nottingham, Fierer, et al., 2018; Fyllas et al., 2017; Rapp et al., 2012; Whitaker et al., 2014; van de Weg, Meir, Grace, & Atkin, 2009).

### 2.2 Soil sampling and analyses

For all sites, soil samples were collected during November 2011. These ecosystems are highly aseasonal, with no significant intra‐annual variation in mean monthly temperature (Rapp & Silman, 2012) and no evidence of seasonal soil or plant moisture constraints (van de Weg et al., 2014; Zimmermann, Meir, Bird, Malhi, & Ccahuana, 2010); therefore, the comparison of soil properties for these sites at a single time point was approximated as representative of patterns likely to be found throughout the year. Furthermore, temperature seasonality has earlier been shown to have no or very little effect on the temperature‐growth response of bacterial communities, even in soils with a large amplitude in temperature over the year (Birgander et al., 2018; van Gestel et al., 2013). We collected soil from four corner subplots and a central subplot, within each of the 1 ha permanent study plots at each elevation site, with soil from these subplots used as five individual replicates. For each subplot, the upper 10 cm surface soil was collected using a soil auger and stored in sealed plastic bags. Soil samples were stored for 1–2 months at approximately 17°C until analysis. Earlier studies have shown that storing soil samples at <25°C for up to 2 months does not affect the temperature characteristics of microbial communities (Bárcenas‐Moreno et al., 2009; Birgander, Reischke, Jones, & Rousk, 2013).

### 2.3 Bacterial and fungal growth

Temperature sensitivity of microbial growth was determined by measuring instantaneous growth of bacteria and fungi at different temperatures, as earlier used by Pietikäinen et al. (2005). Bacterial growth was estimated using the leucine (Leu) incorporation method, while fungal growth was estimated using the acetate‐in‐ergosterol (Ac‐in‐erg)‐incorporation method (Bååth, 2001; Rousk & Bååth, 2011). Since many samples were processed (14 soils × 5 replicates = 70 soil samples), microbial growth was measured for all soils at one temperature on separate days. This experimental design was suitable to determine relative changes in temperature sensitivity with differences in MAT between sites.

The growth rate of bacteria was estimated using the leucine incorporation method, following Bååth, Pettersson, and Söderberg (2001). Briefly, soil samples (1 g fresh weight) were vortexed with 20 ml distilled H_2_O for 3 min. and then centrifuged at 1,000 g for 10 min. Aliquots (1.5 ml) of the resulting suspension were transferred to 2 ml tubes and 2 µl [^3^H]Leu (37 MBq/ml and 5.74 TBq/mmol) combined with unlabelled Leu, resulting in 275 nM Leu in the bacterial suspensions. After incubation at the desired temperature (in water baths), the reaction was terminated with 75 µl 100% trichloroacetic acid. Incubation time was modified according to the incubation temperature to compensate for lower incorporation at low temperatures (Pietikäinen et al., 2005), with 24 hr for 0 and 4°C, and 2 hr for 20 and 35°C. Washing of the samples and measurement of incorporated radioactivity was performed following Bååth et al. (2001).

The growth rate of fungi was estimated using the acetate‐in‐ergosterol‐incorporation method (Newell & Fallon, 1991) adapted for soil (Bååth, 2001), with modifications. Briefly, soil samples (1 g fresh weight) were transferred to test tubes to which 20 µl [1–^14^C] acetic acid (sodium salt; 7.4 MBq/ml and 2.04 GBq/mmol) with unlabelled sodium acetate, and 1.5 ml distilled H_2_O, resulting in a final acetate concentration of 220 µM. The resulting soil slurry was then incubated in the dark (for twice as long as the corresponding samples used for bacterial growth), after which 1 ml formalin (5%) was added to terminate the reaction. Ergosterol was then extracted, separated and quantified using high‐performance liquid chromatography and a UV detector (282 nm). The ergosterol peak was collected, and the amount of incorporated radioactivity was determined.

### 2.4 Calculation of *T*_min_, temperature sensitivity index and *Q*_10_

*T*_min_ was calculated using growth at 4°C and 20°C, assuming a straight‐line relationship for the squared growth rates versus temperature (Figure 1), according to the Ratkowsky equation (Ratkowsky et al., 1982):

$${\text{Growth}}^{0.5}=a\times \left(T-T_{\min}\right)$$

where *T*_min_ (°C) is the apparent minimum temperature for growth, *T* is the measurement temperature (in our case 4 and 20°C), and *a* is a slope parameter related to the absolute growth rate. Since *T*_min_ will always be determined by extrapolation, an alternative temperature sensitivity index, log 35/0 was defined as the log ratio of growth at 35°C and 0°C (Figure 1). A similar ratio was suggested by Bárcenas‐Moreno et al. (2009) as a rapid and sensitive way to study changes in temperature sensitivity and has been shown to correlate with *T*_min_ (Rinnan et al., 2009). We chose a large temperature range for the temperature sensitivity index (i.e., 35°C and 0°C), to accommodate the variation expected by the large range in MAT between sites. Means and *SE* were calculated for each site (*n* = 5 replicate soil samples per site). Regressions against temperature for the different growth indices were then made using mean values per site (*n* = 14), since they were the independent samples. For indices of fungal growth, sample size (*n*) was 13 because one site (TC, high elevation grassland) had activity values which were too low to be able to calculate temperature sensitivity.

We calculated *Q*_10_ for the 10 to 20°C range (*Q*_10–20_) using the mean *T*_min_ values for each site, according to the equation:

$$Q_{R}={\left[\left(T_{L}+R-T_{\min}\right)/\left(T_{L}-T_{\min}\right)\right]}^{2}$$

where *R* = the temperature range (for *Q*_10–20_, *R* = 10) and *T*_L_ is the lowest temperature in the range (e.g., for *Q*_10–20_ *T*_L _= 10) (Bååth, 2018). To calculate *Q*_10_ at MAT±5°C (*Q*_MAT±5_), we modified the equation where *T*_L _= (MAT−5), with MAT from Supporting Information Table S1. To estimate activity with an increase in MAT of 2°C (representing the lowest end of the range of predicted global MAT increase by 2100; 2–6°C; IPCC, 2013), we used *R* = 2 and *T*_L _= MAT. We further modified Equation ([Link]) to account for temperature adaptation—according to our finding that thermal adaptation of growth led to an increase in *T*_min_ by 0.6°C per 2°C increase in MAT—by replacing *T*_min_ with (*T*_min _+ 0.6).

## 3 RESULTS

### 3.1 Soil properties

Increased elevation was highly negatively correlated with a decrease in MAT (*R* ^2 ^= 0.99, *p* < 0.001; Supporting Information Figure S1), such that elevation and MAT were interchangeable as explanatory variables. Increased elevation was associated with an increase in total C (*R* ^2 ^= 0.46, *p* = 0.008) and total *N* (*R* ^2 ^= 0.51, *p* = 0.004); small but nonsignificant increases in total P (*R* ^2 ^= 0.18, *p* = 0.13) and extractable P (*R* ^2 ^= 0.22, *p* = 0.09). Soil pH did not vary with elevation (*R* ^2 ^= 0.001, *p* = 0.90) (Supporting Information Table S1). Further detail on soil and microbial community properties for these sites, including in organic horizons, is provided elsewhere (Nottingham, Turner, et al., 2015; Whitaker et al., 2014).

### 3.2 Bacterial growth

**Figure 2:**
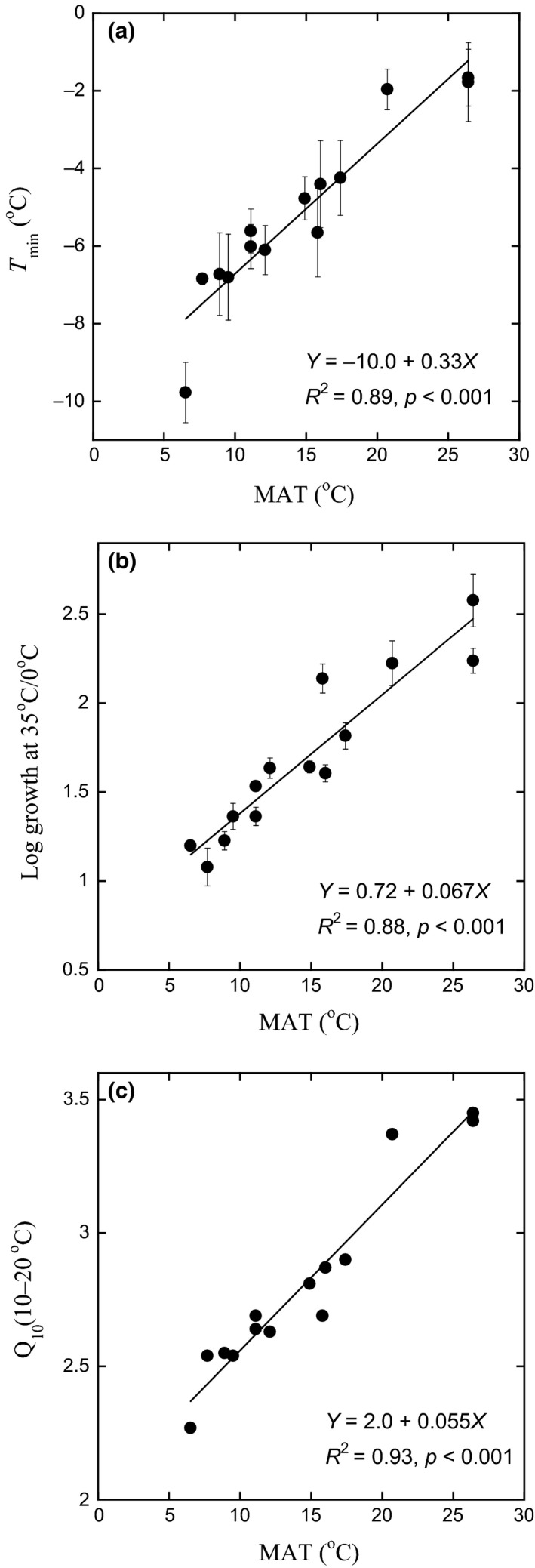
Bacterial community growth response to differences in mean annual temperature (MAT) along an elevation gradient in the Andes. The temperature sensitivity was expressed using three different metrics. (a) Temperature sensitivity expressed by *T*_min_ as affected by MAT, calculated from the Ratkowsky model, (b) temperature sensitivity expressed by the log of the ratio of instantaneous growth at 35/0°C as affected by MAT, (c) temperature sensitivity expressed by *Q*_10–20 _(see Methods) as affected by MAT, calculated from *T*_min_ values. Bars indicate *SE* (*n* = 5). Regressions were calculated with mean values for each site (*n* = 14)

Higher MAT (decreased elevation) resulted in bacterial communities with growth adapted to higher temperatures. All three methods of expressing temperature adaptation of bacterial growth were highly positively correlated with MAT (Figure 2). *T*_min_ varied with MAT according to the equation *T*_min _= −10.0 + 0.33*MAT (*R* ^2 ^= 0.89, *p* < 0.001, Figure 2a). *T*_min_ increased from −8°C at the highest elevation sites with MAT around 6°C to a *T*_min_ of −1.5°C in the lowland sites with MAT around 26°C. This is equivalent to an increased *T*_min_ of 0.33 ± 0.035°C per 1 degree of increase in MAT in this temperature range. In addition, the temperature sensitivity index log 35/0 was highly positively correlated with MAT (*R* ^2 ^= 0.88, *p* < 0.001), also indicating consistent changes in temperature adaptation with changes in MAT (Figure 2b). Given that *Q*_10_ varies with the temperature range used for its calculation (Bååth, 2018; Kirschbaum, 2000), we calculated *Q*_10_ only for one intermediate range of temperatures (between 10 and 20°C). *Q*_10–20_ increased from approximately 2.4 at a MAT of 6°C to almost 3.5 at a MAT of 26°C (*R* ^2 ^= 0.93, Figure 2c). This translates to an increase in *Q*_10–20_ of 0.055 ± 0.004 per 1 degree of increase in MAT for this temperature range.

### 3.3 Fungal growth

**Figure 3:**
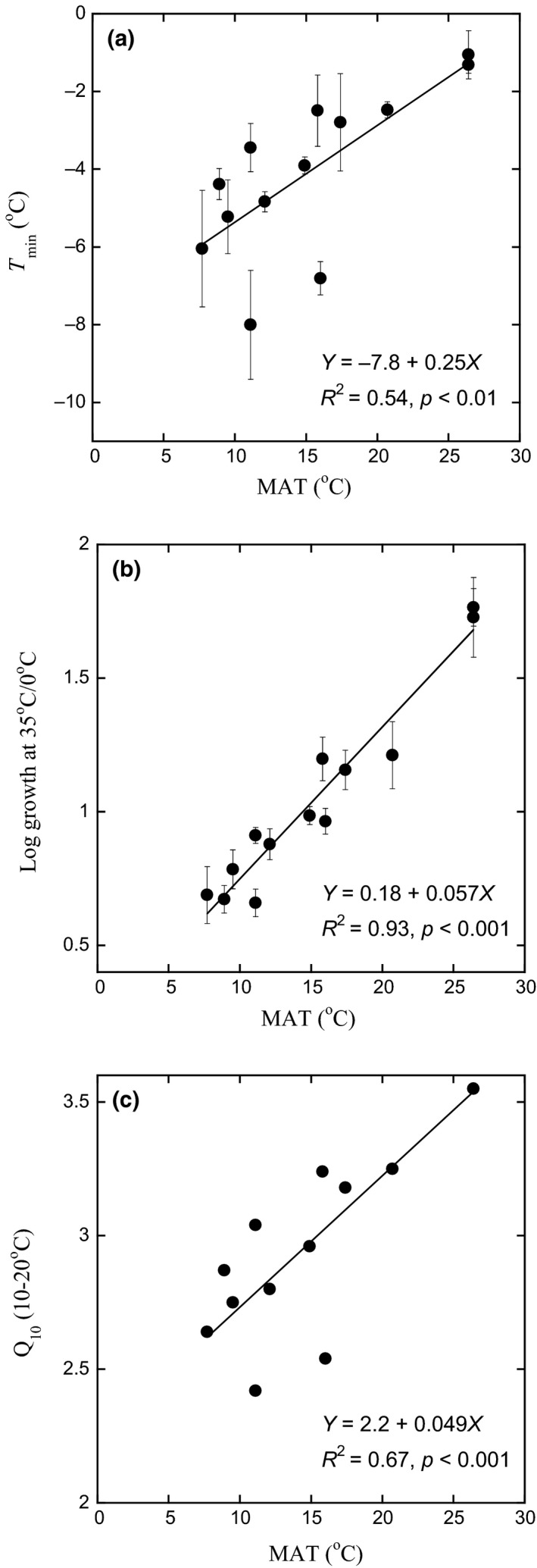
Fungal community growth response to differences in mean annual temperature (MAT) along an elevation gradient in the Andes. The temperature sensitivity was expressed using three different metrics. (a) Temperature sensitivity expressed by *T*_min_ as affected by MAT, calculated from the Ratkowsky model, (b) temperature sensitivity expressed by the log of the ratio of instantaneous growth at 35/0°C as affected by MAT, (c) temperature sensitivity expressed by Q_10–20 _(see Methods) as affected by MAT, calculated from *T*_min_ values. Bars indicate *SE* (*n* = 5). Regressions were calculated with mean values for each site (*n* = 13)

The temperature sensitivity of fungal growth was affected by site MAT in a similar way as bacterial growth (Figure 3). For fungal growth, *T*_min_ varied with MAT according to the equation *T*_min _= −7.8 + 0.25*MAT (*R* ^2 ^= 0.54, *p* < 0.01, Figure 3a). *T*_min_ increased from approximately −6°C in soil from high elevation (MAT ~6°C) to approximately −1°C in soil from low elevation (MAT ~26°C) (*R* ^2 ^= 0.54, *p* < 0.01, Figure 3a). This translates to an increase in *T*_min_ of 0.25 ± 0.069°C per 1 degree of increase in MAT, for this MAT range. The same pattern of fungal growth adapted to MAT was also shown by increases in the temperature sensitivity index (log growth 35/0) and *Q*_10–20_ (*R* ^2 ^= 0.67, *p* < 0.001, Figure 3c) with increased MAT (*R* ^2 ^= 0.93, *p* < 0.001, Figure 3b). The *Q*_10–20_ for fungal growth increased by 0.049 ± 0.0104 per 1 degree of increase in MAT.

### 3.4 Fungal/bacterial relationships

**Figure 4:**
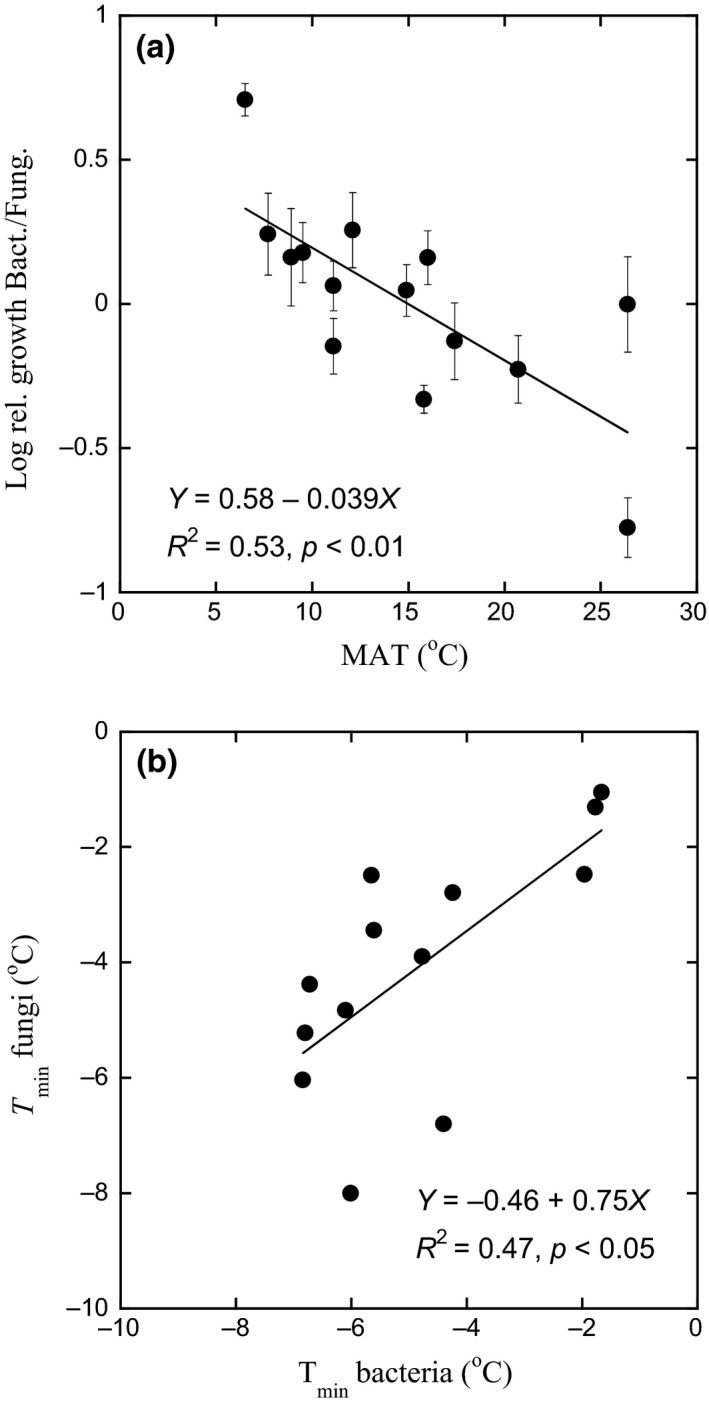
Comparison of the temperature responses of bacterial and fungal growth along an elevation gradient in the Andes, as affected by differences in mean annual temperature (MAT). (a) Relative bacterial to fungal growth. Growth of bacteria was estimated as leucine incorporation and for fungi as acetate‐in‐ergosterol‐incorporation method at 15°C. The data were normalized to a log value of 0 at MAT of 15°C. Bars indicate *SE* (*n* = 5). Regressions were calculated with mean values for each site (*n* = 14). (b) Correlation between *T*_min_ for bacterial and fungal growth

There was a significant decrease in the log ratio of bacterial to fungal growth with increased MAT (*R* ^2 ^= 0.53, *p* < 0.01, Figure 4a), with the ratios approximately five times lower in soil from low elevation (high MAT) compared to soil from high elevation (low MAT). The *T*_min_ for fungal and bacterial growth was linearly related with no significant difference from a 1:1 line (*R* ^2 ^= 0.47, *p* < 0.05, Figure 4b), indicating that fungal and bacterial community responses were similar over the gradient in MAT studied here (6–26°C). This similarity was further indicated by nonsignificant differences between bacteria and fungi for changes in *T*_min_ and Q_10–20 _per 1 degree of increase in MAT.

### 3.5 Predicting future changes

**Figure 5:**
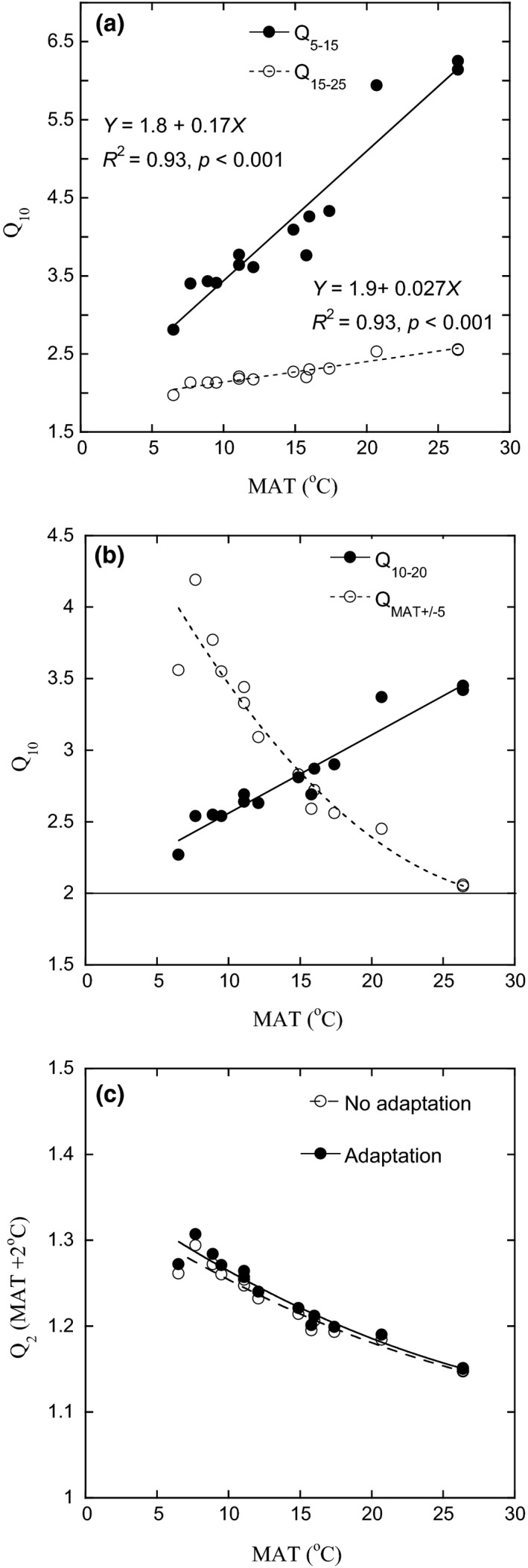
The effect of using different temperature ranges, according to MAT differences along an elevation gradient, to calculate *Q*_10_ values for bacterial growth. (a) *Q*_10_ calculated using *T*_min_ from Figure 2a in the range 5–15°C and 15–25°C. (b) Standardized *Q*_10_ (10–20°C) calculated using *T*_min_ from Figure 2a, compared with in situ specific *Q*_10_ (MAT ±5°C; *Y* = 5.2–0.21*X *+ 0.0033*X* ^2^, *R* ^2 ^= 0.92). The thin line at a value of 2 for *Q*_10_ indicates an approximately upper limit for *Q*_10_ for most enzyme activities (*V*_max_) for these sites (Nottingham et al., 2016). (c) Predicted increases in growth with a 2°C increase in MAT calculated using *T*_min_ from Figure 2a. “No adaptation” was calculated using Equation ([Link]), while “adaptation” was calculated assuming a 0.6°C increase in *T*_min_

We compared *Q*_10_ for the three temperature intervals 5–15°C, 10–20°C and 15–25°C, by using the variation in *T*_min_ for bacterial growth and the square root equation. While *Q*_10_ for 15–25°C only varied between 2.0 and 2.6, *Q*_10_ for 5–15°C varied between 2.8 and 6.3 (Figure 5a) and *Q*_10_ for 10–20°C varied between 2.3 and 3.4 (Figure 2c). Thus, the estimated *Q*_10_ value varied both according to the temperature interval used in the calculation and according to differences in MAT. This was illustrated by comparing *Q*_10_ calculated for a fixed interval (*Q*_10–20_) with *Q*_10_ calculated for MAT±5°C (*Q*_MAT±5_). When calculated over fixed interval, *Q*_10–20_ increased linearly with MAT, indicative of increased temperature sensitivity with increased MAT (Figure 5b using the line in Figure 2c). However, *Q*_MAT±5_ followed the opposite pattern and decreased with increased MAT. For example, for the four highest elevations (MAT ranging from 6.5 to 9.5°C), *Q*_10–20_ values were approximately 2.5, while *Q*_MAT±5_ values were much higher, ranging from 3.5 to 4 (Figure 5b). The opposite pattern was found at lower elevations with higher MAT: In the two lowland forest sites (MAT = 26°C), *Q*_10–20_ values (~3.5) were higher than *Q*_MAT±5_ values (~2). A *Q*_10_ value calculated over a fixed interval therefore gave opposing results when compared to a *Q*_10_ relevant for MAT (e.g., *Q*_MAT±5_).

We used data from Figure 2a to predict the increase in microbial activity with 2°C of warming (Figure 5c). Assuming no adaptation, where *T*_min_ does not respond to warming, the increase in microbial activity with warming was largest in soils from high elevation and low MAT. For example, microbial activity was predicted to increase by 27% in the four sites with lowest MAT and by 15% in the sites with highest MAT. When we accounted for an adaptation response, whereby *T*_min_ increased by 0.6°C per 2°C increase in MAT (Figures 2 and 3a), these predicted increases in microbial activity with warming were slightly higher, with the largest increases for soils from high elevation (low MAT). For example, the predicted increase in “temperature‐adapted” soil microbial activity with a 2°C warming was 28% in the four sites with lowest MAT (an increase of 1.2% points compared to no adaptation), while at a MAT of 26°C, the predicted increase was only 15% (an increase of 0.3% points compared to no adaptation; Figure 5c).

## 4 DISCUSSION

### 4.1 Adaptation to MAT along the gradient

Our main finding, along the 3.5 km tropical elevation gradient with a 20°C change in MAT, was that the temperature response of microbial growth (*T*_min_) is determined by MAT. Our results suggest that an increase in MAT by 1°C will result in an increased *T*_min_ by approximately 0.3°C (and *Q*_10–20_ by 0.05 units) for bacteria and fungi. This outcome is consistent with our second hypothesis. This also provides further evidence for long‐term temperature adaptation of soil microbial growth (hypothesis 1) and the first information for both bacteria and fungi across such a large MAT range and in the tropical biome.

Our key finding that the long‐term temperature adaptation of microbial growth results in a 0.3°C increase in *T_min_* per 1 degree of increase in MAT is consistent with studies of the temperature response of bacteria performed in other ecosystems, although over much narrower ranges in MAT. For example, *T*_min_ of bacterial growth increased by 0.24–0.38°C per 1°C MAT increase along a 13°C MAT gradient in Antarctica (−4 to 9°C) (Rinnan et al., 2009), while 3 years of experimental soil warming (+5°C) in a temperate forest with a MAT of 7°C increased the *T*_min_ of bacterial growth by 0.19°C per 1°C warming (Rousk et al., 2012). A recent compilation of studies on the temperature adaptation of bacterial growth found the same pattern we show here: on average *T*_min_ increased by approximately 0.3°C per 1 degree Celsius increase in MAT (Bååth, 2018). Thus, our findings extend previous observations for bacteria and fungi and across a large MAT range of 6–26°C; in particular, our data fill the gap in understanding for the 9–26°C MAT range, on the thermal adaptation of soil microbial growth to differences in MAT.

Although this is the first study in which the temperature adaptation (*T*_min_) of soil bacterial growth has been evaluated in tropical ecosystems, our estimates of the absolute value of *T*_min_ are consistent with findings from other ecosystems with similar MAT. For example, we found a *T*_min_ of bacterial growth of approximately −8°C at the highest elevation sites with MAT of 6.5°C, which is similar to a *T*_min_ of bacterial growth of −5 to −8°C for several sites in southern Sweden with MAT of approximately 8°C (Dıaz‐Raviña et al., 1994; Pietikäinen et al., 2005; Rinnan et al., 2009), but lower than a *T*_min_ for bacterial growth of −11°C for Antarctic soils with MAT of −4°C (Rinnan et al., 2009). In a desert soil with mean seasonal temperature of 27°C, the *T*_min_ for bacterial growth ranged between −1 and 0°C (van Gestel et al., 2013), consistent with a *T*_min_ of −1.5°C in our lowland forest sites with MAT of approximately 26°C. These consistencies among studies spanning humid tropical forest, dry desert, temperate and Antarctic ecosystems together suggest a very generally applicable finding: The *T*_min_ of microbial growth is strongly determined by ambient temperature regimes and is not constrained by differences in other climatic or edaphic factors. This was also suggested in a tentative global envelope of *T*_min_ for soil microbial activity and growth proposed by Bååth (2018), with cold, polar regions having *T*_min_ between −10 and −15°C, temperate regions (including the high elevation sites in the present study) between −5 and −10°C and warm, tropical regions (including our low elevation sites) having *T*_min_ between 0 and −5°C. Our results, covering such a large span of MAT, thus strongly corroborate the global variation in *T*_min_ hypothesized by Bååth (2018).

### 4.2 Comparing temperature effects on bacterial and fungal growth

Our results showed that bacterial growth and fungal growth respond similarly to temperature differences, contrary to our third hypothesis that fungi would be better adapted to lower temperatures (have a lower *T*_min_). The ranges of *T*_min_ for fungi (−1 to −6°C) and bacteria (−1.5 to −8°C) were similar, and the relationships between *T_min_* and MAT difference were not significantly different between the two microbial groups. This finding contrasts with the study by Pietikäinen et al. (2005), where a lower *T*_min _for fungi was found in comparison with bacteria in a study of two soil types, suggesting increased dominance of fungi during cold periods. Based on our more comprehensive data from 14 different soils, our results run counter to the hypothesis that fungi have a lower *T*_min_ compared to bacteria. Our data on the ratio of bacterial/fungal growth (Figure 4a) showed relatively more bacterial than fungal growth at lower MAT in the highland soils. Our results might thus suggest that earlier studies indicating fungal dominance in cold environments may be explained by other environmental factors covarying with temperature (e.g., *N* availability; Nottingham, Hicks, et al. (2018); Nottingham, Turner, et al. (2015)). A further complicating factor is that the methodology provides proxies for bacterial and fungal growth and there may be small methodological errors when comparing results for bacterial growth (method reflects protein synthesis) with results for fungal growth (method reflects membrane synthesis). As discussed by Pietikäinen et al. (2005), these methodological differences could affect the determined *T*_min_ of growth, but the effect is likely to be minor. Furthermore, it has been shown that small variations in *T*_min_ have only minor effects on predicted yearly activities (Rousk et al., 2012).

### 4.3 Application of *T*_min_ and the square root equation for respiration and growth

In studies of respiration along the same elevation transect, *Q*_10–20_ of soil respiration varied between 2.1 and 6.9 (Zimmermann, Meir, Bird, Malhi, & Ccahuana, 2009), which is equivalent to a *T*_min_ variation of −12.3 to +3.9°C, assuming a square root relationship (equation 4 in Bååth, 2018). This is a similar range, albeit slightly larger, to that found for microbial growth. However, the variation in *Q*_10_ for respiration was not related to elevation (Zimmermann et al., 2009), suggesting no temperature adaptation of respiration. However, only four sites were studied in Zimmermann et al. (2009), with large variations in estimates of *Q*_10_ of soil respiration, which likely reflected different temperature responses of soil and root‐derived respiration (Zimmermann et al., 2010). Bååth (2018) argued, using a compilation of a large number of respiration studies (Hamdi, Moyano, Sall, Bernoux, & Chevallier, 2013) and models used to predict respiration (Del Grosso et al., 2005; Jenkinson et al., 1991; Kätterer & Andren, 2001; Kirschbaum, 2000; Lloyd & Taylor, 1994; Svensson et al., 2008), that *T*_min_ (and *Q*_10_) for microbial growth and respiration should be very similar in soils globally, and that both could be described by the square root equation. Although more precise data on respiration–temperature relationships for the present elevation gradient are needed, we suggest that our data on the temperature sensitivity of soil microbial growth will also be relevant for respiration. Specifically, we hypothesize that our result showing that an increase in MAT by 1°C results in an increased *T*_min_ by approximately 0.3°C (and *Q*_10–20_ by 0.05 units) over the gradient of MAT between 6 and 26°C may also be applicable for soil heterotrophic respiration.

The application of the square root model (using *T*_min_) was suggested as a simple method to quantify the growth or activity response of the microbial community to the temperature regime (Bååth, 2018). This approach is particularly useful because, unlike *Q*_10_, *T*_min_ values are not dependent on the temperature range used for their calculation. This was clear in the present study. Thus, the use of one *Q_10_* value for the range of temperatures found in the present elevation gradient would result in bias when predicting growth and respiration. In contrast, *T*_min_ as descriptor of temperature adaptation of the community and the square root model to estimate the temperature response can be used to calculate *Q*_10_ for any temperature interval (Bååth, 2018). Thus, *T*_min_ can be used to calculate a standardized *Q*_10_ in each of the sites studied, for example, *Q*_10–20_ in Figure 2c, but also to calculate a *Q*_10_ related to the MAT at each specific site (Figure 5b).

### 4.4 Comparing temperature sensitivity of growth and enzyme activity

Before microorganisms can use soil organic matter for growth and respiration, macromolecules must be degraded by extracellular enzymes. The strong temperature‐adaptive responses of microbial growth we found across this elevation and MAT gradient (Figures 2 and 3c) occurred despite a largely insensitive temperature response of enzyme activities reported in a previous study of the same gradient (Nottingham et al., 2016). This previous study of enzyme temperature sensitivity found no elevation patterns in the *Q*_10_ of the maximum enzymatic catalytic rate (*V*_max_) for 5 out of 7 soil enzymes, with only small increases in *Q*_10_ for *V*_max _with increased elevation for 2 enzymes, ß‐glucosidase and ß‐xylanase (Nottingham et al., 2016). Studies from other sites are consistent with a general insensitivity of *Q*_10_ of enzymatic *V*_max _to temperature. Nine years of experimental soil warming in a temperate forest increased enzymatic *V*_max_ but did not affect its* Q*_10_ response (Schindlbacher, Schnecker, Takriti, Borken, & Wanek, 2015); while a cross‐latitudinal study found no differences in the *Q*_10_ of *V*_max _for five hydrolytic enzymes, although a relationship was observed between MAT and the *Q*_10_ of the half‐saturation constant (*K*_m_) of ß‐glucosidase (German, Marcelo, Stone, & Allison, 2012). Thus, the temperature responses of growth do not appear to be principally the result of differences in enzyme function, and there appear to be differences both in the applicable model and in the adaptation responses, for enzyme activity, growth and respiration. Enzymatic activity usually follows a strict Arrhenius relationship with temperature (Davidson, Janssens, & Luo, 2006; German et al., 2012), with very little increase in *Q*_10_ at decreasing temperature. Furthermore, *Q*_10_ values of enzyme activity are often ≤2, irrespective of temperature (Nottingham et al., 2016; Schindlbacher et al., 2015). A comparison of the temperature sensitivities (*Q*_10_) of MAT‐relevant microbial growth and enzyme activities (Figure 5b), indicates that enzyme activities have a lower *Q*_10_ over the whole range of MAT, with the discrepancy increasing at lower temperatures. Overall, these results reinforce the need to understand intrinsic temperature responses of discrete biochemical processes—microbial growth, respiration and enzymatic activity—which together determine the temperature response of the overall C balance (Conant et al., 2011; Davidson et al., 2006).

### 4.5 Modelling adaptation: using *Q*_10_ calculated over a fixed interval and at MAT

We show that a *Q*_10_ value calculated using MAT (*Q*_MAT±5_) provides a robust metric to model temperature responses, but a *Q*_10_ value calculated over a fixed interval (e.g., *Q*_10–20_) gives misleading results when comparing sites with differences in MAT. The accurate estimation of *Q*_MAT±5_ was possible for the studied elevation gradient, because there is little annual and seasonal variation in temperature at each site (Rapp & Silman, 2012; Zimmermann et al., 2010). In sites with low MAT, *Q*_MAT±5_ was higher than *Q*_10–20_ and *vice versa* in sites with high MAT (and *Q*_MAT±5_ = *Q*_10–20_ where MAT = 15°C). This resulted in a *Q*_10–20_ that increased with MAT (2.3 at 6°C to 3.4 at 26°C), but a *Q*_MAT±5_ that decreased with MAT (3.7 at 6°C to 2 at 26°C). Thus, *Q*_10–20_ (or determined across any fixed temperature interval) is useful to compare the relative temperature responses of different processes among studies across the same temperature range, while *Q*_MAT±5_ is useful for modelling temperature responses across gradients in MAT. However, *Q*_10–20_ is misleading where temperature ranges differ among studies, and it is difficult to use this information to infer general responses to future climate warming across different biomes.

**Figure 6:**
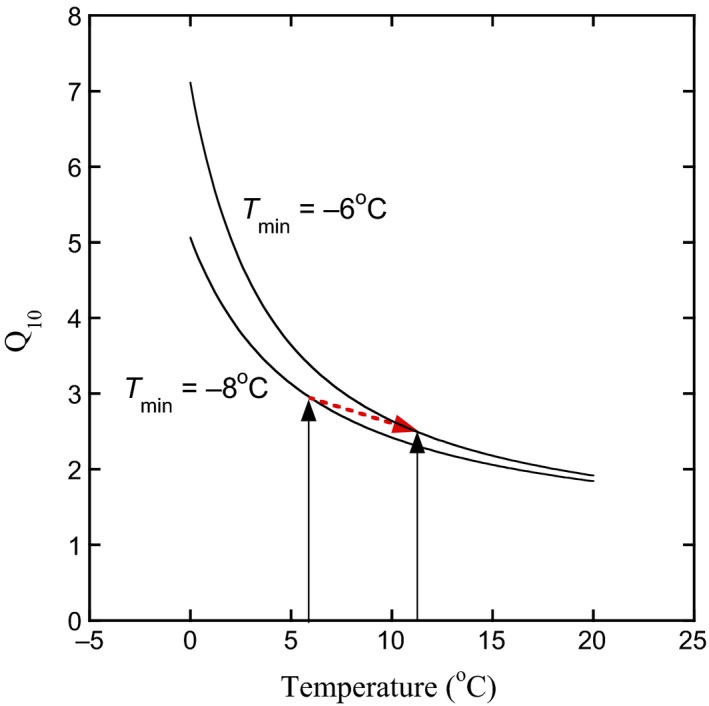
Conceptual figure on the effect of increasing MAT on *Q*_10_, calculated using the square root relationship. A soil with MAT of 6°C, and a *T*_min_ of −8°C, has *Q*_10_ ~3 (left black arrow). By increasing MAT by 6°C to 12°C (right black arrow), there is a decrease in *Q*_10_ (~2.7) but an increase in *T*_min_ (by 2°C to −6°C). The new *T*_min_ trajectory results in a decrease in *Q*_10_ when calculated at the new temperature (*Q*_MAT+2_; red dashed arrow) but a higher *Q*_10_ if calculated across a fixed temperature range (e.g., *Q*_0–10_). The trajectories were calculated using the square root relationship for *T*_min_ of −6 and −8°C (see Bååth, 2018) [Colour figure can be viewed at wileyonlinelibrary.com]

Thus, using this model based on *Q*_MAT±5_, we can conceptualize the microbial growth response to warming as the result of two counteracting effects: the direct temperature effect according to the *Q*_10_ trajectory at a fixed *T*_min_ and the adaptation effect in changing the *T*_min_ (and thereby altering the *Q*_10_ trajectory; Figure 6). Using data from Figure 2a for bacterial growth, a soil with MAT of 6°C will have a *T*_min_ of −8°C and *Q*_10_ will vary with temperature according to the −8°C trajectory: decreasing with increasing temperature. However, we can include our results for long‐term temperature adaptation of microbial growth, 0.3°C increase in *T*_min_ per degree increase in MAT (Figures 2 and 3). By including temperature adaptation in this model, +6°C warming will increase *T*_min_ by around 2°C and alter the *Q*_10_ trajectory to one where microbial growth is slightly higher at the new temperature regime (dashed red arrow; Figure 6).

### 4.6 Predicting effects of future climate change scenarios

The use of *T*_min_ and the square root equations will enable simple estimation of the temperature sensitivity across the MAT range relevant for future predicted climate change scenarios. Cramer *et al*. (2004) predicted a warming in the tropics of 4° by 2100; this could be modelled by calculating *Q*_4_ for MAT+4°C. A similar calculation was made for heterotrophic respiration at site‐specific MATs by Zimmermann et al. (2009) for four of the sites (at 3,030, 1,500, 1,000 and 200 m elevation equivalent to 11°C to 26°C MAT), with *Q*_4_ relevant to climate change predictions estimated to be 1.66, 1.29, 1.27 and 1.0 (i.e., with an increasing MAT of 4°C). Calculating similar *Q*_4_ values for bacterial growth and MAT +4°C resulted in very similar predictions, 1.55, 1.40, 1.38 and 1.31. These similarities are thus further indications that our estimates of temperature sensitivity of microbial growth are also relevant for heterotrophic respiration.

The predicted global warming by 2100 ranges from 1.4 to 5.8°C, based on a range of emission scenarios (IPCC, 2013). Thus, considering a conservative 2°C increase in global MAT, the relative impact on microbial activity (growth and respiration) will be stronger in ecosystems with lower MAT (28%) than with higher MAT (15%), suggesting that with the same predicted increase in MAT, the relative effect will be stronger at lower temperatures. The relatively greater impact at lower MAT may be further exacerbated because greater warming is predicted in higher‐latitude ecosystems (IPCC, 2013), although significant impacts in tropical regions could occur if MAT exceeds thermal optima for activity (*T*_opt_).

Because the elevation gradient under study here is largely aseasonal in temperature (Rapp & Silman, 2012), we could use MAT and one single *Q*_MAT±5_ to characterize the temperature adaptation of microbial growth. This will not be the case in ecosystems with large seasonal temperature variation, including deserts (van Gestel et al., 2013) or temperate and continental climates, where *Q*_10_ will vary seasonally (Bååth, 2018). However, by determining *T*_min_ and using the square root equation, it is straightforward to model the effect of seasonal temperature variation, as shown by Rousk et al. (2012). Similar to our study, their data also suggested that the effect of temperature adaptation was minor compared to the effect of seasonal temperature variation, in determining the *Q*_10_ of microbial activity (cf Figure 5c,6).

Our results demonstrate consistent patterns of temperature adaptation (*T*_min_) in growth across a large temperature range. Our results also show how *T*_min_ can be used as a single descriptor of temperature adaption of the microbial community, which together with the square root equation (Ratkowsky et al., 1982) can be used to predict temperature effects on microbial growth. However, in order to fully understand climate warming impacts on microbial communities and the carbon balance, further studies are required on the responses of microbial carbon use efficiency (carbon uptake, growth and respiration) (Bradford, 2013) and on the intrinsic temperature responses of, and the interactions between, different physical, biological and chemical components of the soil carbon cycle (Conant et al., 2011; Nottingham, Turner, et al., 2015). A major outstanding question is also whether these microbial growth responses to long‐term temperature differences observed along a tropical mountain elevation gradient, will shift either through acclimation or adaptation in response to short‐term climatic change.
