# Supplementary material for: Adaptation of soil microbial growth to temperature: Using a tropical elevation gradient to predict future changes
Source: Glob Chang Biol. 2019 Jan 6;25(3):827–38. doi: 10.1111/gcb.14502 (PMC6392126; doi:10.1111/gcb.14502)
Supplement: Supplementary file 1 [file GCB-25-827-s001.docx]

**Adaptation of soil microbial growth to temperature: using a tropical elevation gradient to predict future changes**

**Nottingham AT, Bååth E, Reischke S, Salinas N, Meir P**

**Supporting Information**

**Table S1**

**Figure S1**

**Table S1. Climatic and soil properties for 14 sites along a 3.5 km elevation gradient in the Peruvian Andes.** Data are mean annual temperature (MAT), mean annual rainfall (MAP) and soil properties of pH, total elements (CNP) and resin-extractable P (ext P).

| Plot code | elevation (m asl) | MAT (^o^C) | MAR (mm) | pH | C  (%) | N  (%) | P  (mg P g^-1^) | | ext P  (mg P kg^-1^) |
| --- | --- | --- | --- | --- | --- | --- | --- | --- | --- |
| TAM4 | 194 | 26.4 | 2730 | 4.6 | 2.38 | 0.35 | 0.49 | 3.3 | |
| TAM3 | 210 | 26.4 | 3199 | 3.8 | 1.70 | 0.23 | 0.18 | 2.7 | |
| VC | 1000 | 20.7 | 3087 | 3.8 | 16.2 | 1.34 | 0.73 | 0.7 | |
| SP2 | 1500 | 17.4 | 2631 | 4.0 | 10.3 | 0.91 | 1.36 | 44.7 | |
| SP1 | 1750 | 15.8 | 2631 | 3.9 | 26.0 | 1.56 | 1.44 | 19.0 | |
| TU8 | 1850 | 16.0 | 2472 | 3.9 | 31.1 | 1.86 | 0.76 | 14.4 | |
| TU7 | 2020 | 14.9 | 1827 | 4.0 | 37.0 | 2.00 | 0.71 | 16.3 | |
| TU5 | 2520 | 12.1 | NA | 3.9 | 25.8 | 1.73 | 0.98 | 53.1 | |
| TU4 | 2720 | 11.1 | 2318 | 3.9 | 28.6 | 1.64 | 0.87 | 56.0 | |
| TU3 | 3020 | 9.5 | 1776 | 3.8 | 27.1 | 1.57 | 0.92 | 59.7 | |
| WAY | 3025 | 11.1 | 1706 | 4.1 | 46.5 | 2.39 | 1.09 | 82.0 | |
| TU2 | 3200 | 8.9 | NA | 4.1 | 44.8 | 2.42 | 0.91 | 72.8 | |
| TU1 | 3400 | 7.7 | 2555 | 4.0 | 42.1 | 2.49 | 1.09 | 223.5 | |
| TC | 3644 | 6.5 | NA | 4.9 | 23.7 | 1.60 | 0.89 | 2.52 | |

**Fig. S1. Correlation between Mean Annual Temperature (MAT) and elevation, along an elevation gradient in the Andes.**
